# Antibody-based sex determination of human skeletal remains

**DOI:** 10.1016/j.isci.2023.108191

**Published:** 2023-10-12

**Authors:** Barry Shaw, Sophie Foggin, Petter Hamilton-Stanley, Andy Barlow, Catriona Pickard, Linda Fibiger, Neil Oldham, Patrick Tighe, Lisette M. Kootker, Sarah Schrader, Rob Layfield

**Affiliations:** 1 School of Life Sciences, University of Nottingham, Nottingham, UK; 2 School of History, Classics and Archaeology, University of Edinburgh, Edinburgh, UK; 3 School of Chemistry, University of Nottingham, Nottingham, UK; 4 Department of Earth Sciences, Vrije Universiteit Amsterdam, Amsterdam, the Netherlands; 5 Faculty of Archaeology, Leiden University, Leiden, the Netherlands

**Keywords:** Immunology, Methodology in biological sciences, Paleobiology

## Abstract

Assignment of biological sex to skeletal remains is critical in the accurate reconstruction of the past. Analysis of sex-chromosome encoded AMELX and AMELY peptides from the enamel protein amelogenin underpins a minimally destructive mass spectrometry (MS) method for sex determination of human remains. However, access to such specialist approaches limits applicability. As a convenient alternative, we generated antibodies that distinguish human AMELX and AMELY. Purified antibodies demonstrated high selectivity and quantitative detection against synthetic peptides by ELISA. Using acid etches of enamel from post-medieval skeletons, antibody determinations corrected osteological uncertainties and matched parallel MS, and for Bronze Age samples where only enamel was preserved, also matched MS analyses. Toward improved throughput, automated stations were applied to analyze 19th-century teeth where sex of individuals was documented, confirming MS can be bypassed. Our immunological tools should underpin development of routine, economical, high-throughput methods for sex determination, potentially even in a field setting.

## Highlights

• Antibodies can distinguish human sex-chromosome encoded AMELX and AMELY peptides
• Immunoassays using dental enamel extracts allow sex determination of human remains
• Use of antibodies shoud allow sex determination to become a more routine activity

## Graphical abstract

**Figure:**
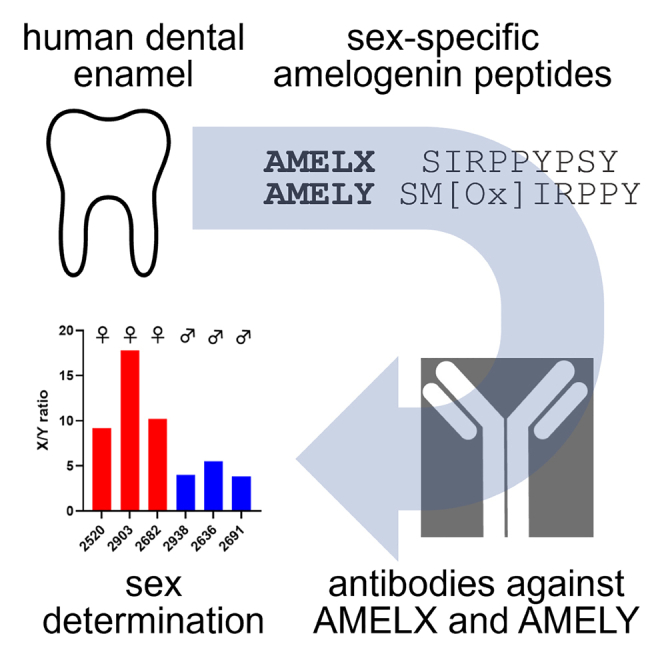

## Introduction

Analysis of ancient skeletal remains can provide information related to the historical and cultural contexts of the sample(s) through a variety of techniques, with conventional osteological determinations to determine biological sex in adults requiring ideally the presence of pelvic bones and skulls. In contrast, other approaches such as ancient DNA (aDNA) analysis are more destructive and rely on access to specialist approaches with equipment and facilities, and depend on well preserved bones (pars petrosa, long bones) or teeth (dentine). Where skeletal remains are poorly preserved, often only dental enamel remains, which in recent years have been realized to be a rich source of preserved ancient peptides and proteins that are amenable to proteomic analysis.

Sex determination of human skeletal remains informs about past societies and their demographics and, equally, can identify missing individuals in forensic contexts. Sex estimation of adult skeletons using sexually dimorphic characteristics of the pelvis and cranium can be challenging^1^ and is further complicated for pre-pubescent individuals due to limited hormone differentiation.^2^ Archaeological remains may be incomplete, found in a non-organized manner, and affected by taphonomic or diagenetic processes, further complicating osteological analyses. To complement osteological analyses, biochemical, immunological, and genetic approaches have been developed, with aDNA analysis not only able to determine biological (chromosomal) sex but also identify and classify unknown hominins,^3^ aid species identification,^4^ and to establish kinship between individuals and reconstruct phenotypes.^5^ However, aDNA is susceptible to degradation in older samples, and indeed younger samples dependent on soil conditions, with contamination from contemporary DNA a potential caveat.

In contrast, ancient proteins may in certain circumstances be better preserved, including in dental enamel recovered in archaeological excavations, and proteomic analyses are not reliant on amplification steps which can also amplify modern contaminants. Indeed, some proteins can survive in calcified materials for millions of years^6^ with earlier work indicating the possibility of using immunological approaches for detection and species attribution of protein extracted from fossil bone dated at dated at 1.6 Myr.^7^ Proteomic approaches have been developed around the use of mass spectrometry (MS) to detect ancient proteins. In particular, zooarchaeology by MS (ZooMS), identifies species-specific enzymatically derived peptide mass fingerprints from collagen preserved in ancient samples.^8^

Notably, a liquid chromatography MS (LC-MS) approach for human sex determination has been developed, which has the potential to revolutionize bioanthropology.^9^ This approach is based on detection of sex-specific endogenous peptides preserved into deep time in dental enamel, which can be retrieved using minimally destructive acid etching of teeth. The human *AMELX* and *AMELY* genes are located on the X and Y chromosome, respectively,^10^ and give rise to sexually dimorphic amelogenin proteins which are involved in tooth enamel formation.^11^ Amelogenin protein undergoes a natural proteolysis to produce bioactive peptides, with some of these peptides representing sex-specific sequences. Specifically, detection of endogenous peptides denoted AMELX and AMELY using LC-MS, can be used to assign biological sex.^9^ This approach has found prominence in studies of human history, for example clarifying unexpected funerary practices associated with the Late Antique “Lovers of Modena”^12^ and confirming biological sex of ancient hominin (*Homo antecessor*) remains.^13^

Although MS-based detection of AMELX and AMELY peptides offers a significant advance through the availability of a rapid and inexpensive method for sex determination, in practice MS approaches can often be inaccessible to the fields of archaeology and anthropology. Here, we provide a proof-of-concept that simple and routine immunological detection of AMELX and AMELY peptides can bypass the reliance for specialist MS-based approaches, potentially underpinning development of methods that ultimately could be employed in a field setting.

## Results

### Generation of AMELX and AMELY specific antibodies

**Figure 1:**
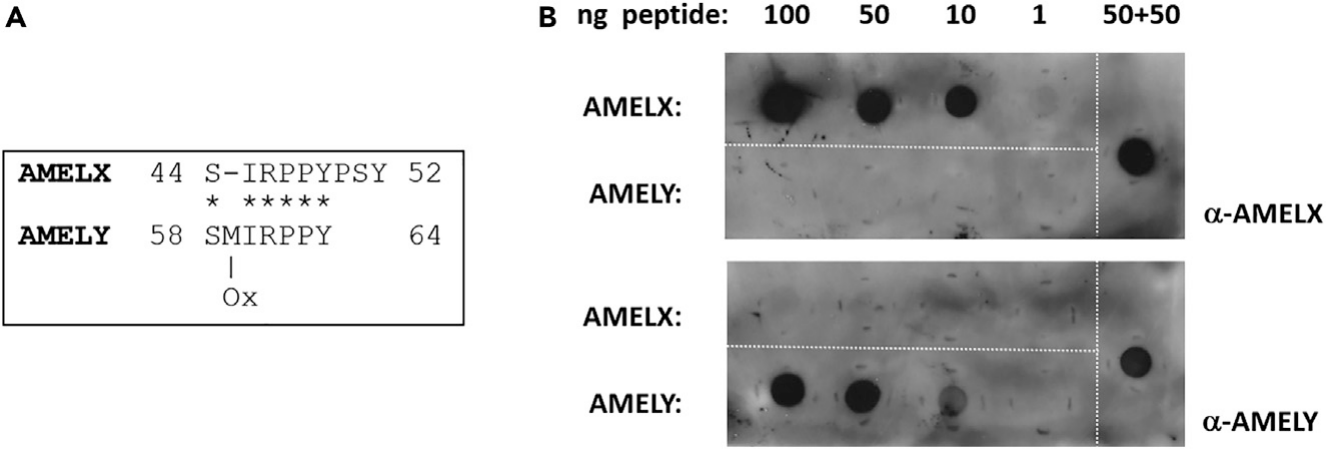
Amelogenin peptide sequences and antibody detection by dot-blot (A) Sequence alignment of endogenous sex-specific human amelogenin peptides AMELX and AMELY. Asterisks indicate sequence identity, “-” indicates gap introduced in the alignment, M[Ox] represents oxidized methionine, numbering relates to amino acid residues in full-length amelogenin sequences. (B) Representative dot-blot indicating affinity purified antibody detection of appropriate synthetic peptides at 10ng sensitivity, with no visual evidence of cross-reactivity of anti-AMELX antibody against AMELY peptide, or vice versa (detection antibodies shown on the right-hand side). “50 + 50” indicates a mixture of AMELX and AMELY peptides.

Toward an alternative approach to existing MS-based detection of endogenous sex-specific human amelogenin peptides, we raised synthetic peptide antibodies against AMELX (sequence SIRPPYPSY) and AMELY (sequence SM[Ox]IRPPY, where M[Ox] represents oxidized methionine; Figure 1A), the same endogenous sexually dimorphic peptides previously established and detected in MS-based sex determinations.^9^ Synthetic peptide antigens retained a free N-terminus as in the endogenous sequence, with an additional C-terminal cysteine residue incorporated to permit conjugation to carrier protein and affinity chromatography matrices. Rabbit polyclonal antibodies were generated (Eurogentec) with final antisera subject to affinity depletion using the “wrong” synthetic peptide (e.g., α-AMELX vs*.* AMELY peptide) and subsequent affinity enrichment using the corresponding antigenic peptide (e.g., depleted α-AMELX vs*.* AMELX peptide).

### Antibody evaluation: Dot-blot

As an initial test of antibody selectivity, purified antibodies were tested by immunoblotting (dot-blot) with aliquots of the same synthetic peptides used for antibody generation utilized as antigens, manually spotted on to nitrocellulose membranes. After incubation with primary antibodies, dot-blots were developed with peroxidase-conjugated secondaries and ECL (enhanced chemiluminescence), and found to detect appropriate peptides at 10ng sensitivity, with no visual evidence of cross-reactivity of anti-AMELX antibody against AMELY peptide, or vice versa (Figure 1B), despite sequence similarities (Figure 1A).

### Antibody evaluation: ELISA

**Figure 2:**
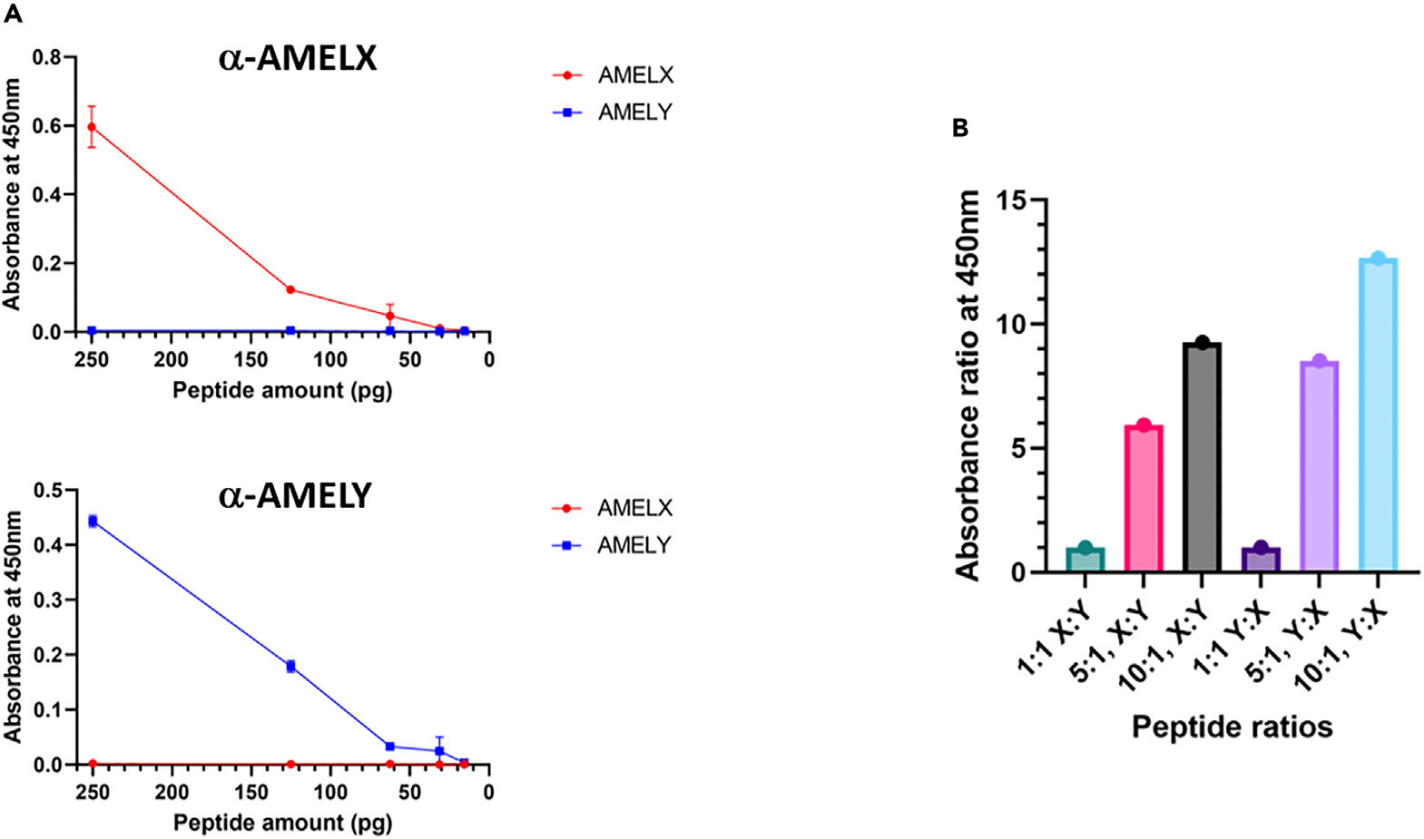
ELISA detection of synthetic amelogenin peptides (A) Representative ELISAs demonstrating appropriate detection of synthetic AMELX (red lines) and AMELY (blue lines) peptides by affinity-purified antibodies as indicated, down to ∼100pg antigen, again with limited cross-reactivity against the “wrong” peptide. Data are represented as mean ± SEM. (B) Bar graph demonstrating the quantitative nature of ELISA allows distinction of mixtures of synthetic AMELX and AMELY peptides at various ratios as indicated (500pg total peptide e.g., 1:1 represents 250pg of each peptide). Y axis indicates ratios of observed ELISA values, determined by separately probing the same peptide mixtures with α-AMELX or α-AMELY antibodies (e.g., for 5:1, X:Y, α-AMELX value divided by α-AMELY value).

To improve sensitivity of detection of AMELX and AMELY peptides by the purified antibodies, optimized conditions for ELISA (Enzyme-Linked Immunosorbent Assay) were determined. Loading of peptide antigens in PBS, with blocking in 5% (w/v) BSA in TBS-Tween, and primary (1:500) and secondary antibodies (1:2000) diluted in the same blocking buffer, reliably allowed appropriate detection of synthetic peptides down to ∼100pg antigen, again with limited cross-reactivity (Figure 2A). Notably, the quantitative nature of ELISA allowed clear distinction of mixtures of synthetic AMELX and AMELY peptides at various ratios (500pg total peptide, Figure 2B), indicating the future potential to probe rare cases of chromosomal abnormalities (see Discussion).

### Analysis of archaeological human teeth

**Figure 3:**
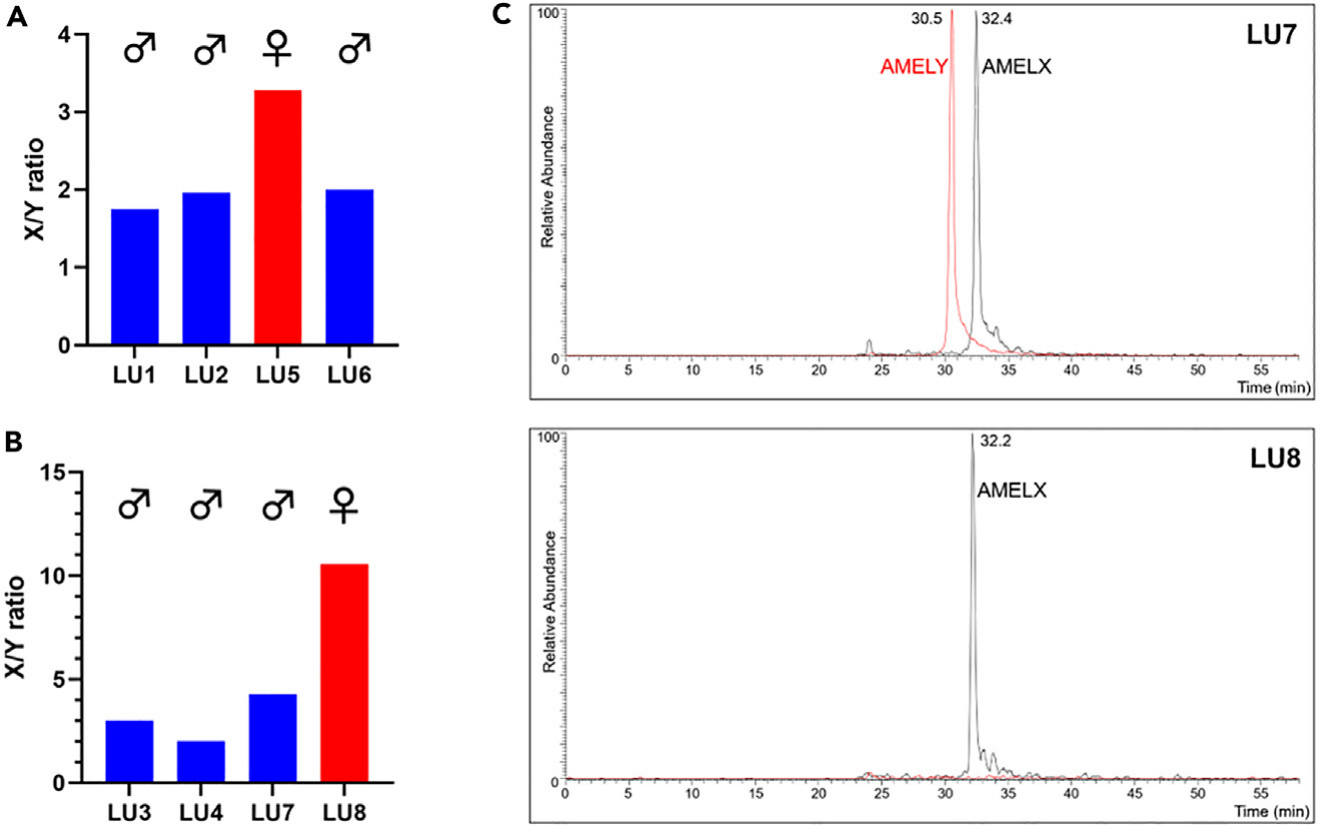
Sex determination of post-medieval human teeth using manual ELISA (A) AMELX/AMELY (X/Y) ratios detected by ELISA for enamel extracts from four post-medieval human teeth (12.5% of each total enamel extract loaded per well, in duplicate). (B) As for (A) with an additional four post-medieval human teeth with lower sample load (6.25% of each total enamel extract loaded per well, in duplicate). Male or female assignments, based on ratios, are indicated. (C) Representative extracted ion chromatograms showing the MS/MS transitions *m/z* 540.3 → 879.5 (AMELX, black) and *m/z* 440.2 → 645.4 (AMELY, red) demonstrating that LU7 and LU8 are samples from a male and a female, respectively.

To assess the ability of antibody-based detection of endogenous AMELX and AMELY peptides in the assignment of biological sex to human samples, the optimized ELISA approach was applied to extracts of dental enamel from eight post-medieval skeletons, blinded against standard osteological estimations, and to MS-based analyses of the same enamel extracts in parallel. Minimally destructive acid etching of whole teeth combined with peptide purification by reverse-phase solid-phase extraction (RP-SPE, C18) was used for sample preparation, essentially according to Stewart et al.^9^ For the first four teeth analyzed, 12.5% of each total enamel extract was loaded per well, with duplicate samples probed by each of the antibodies using the optimized ELISA conditions on the same plate, and with sample detection displayed as AMELX/AMELY (X/Y) ratios (the remaining 50% of sample committed for LC-MS). The ELISA analyses clearly distinguished the single female sample from the three males (Figure 3A) and matched osteological estimations (Table 1). Although for female samples, X/Y ratios should logically tend to infinity (with AMELY not expressed), the observed ratio of 3.28 for the female sample presumably reflects low-level non-specific binding of (α-AMELY) antibodies to components of the enamel extracts. Indeed, for the second four post-medieval teeth, where sample loading was halved to 6.25% of the total enamel extract per well, X/Y ratio for the single female sample increased to > 10 (Figure 3B). For two of these second set of four teeth, LU3 and LU7, ELISA-based sex determinations disagreed with the osteological estimations of probably female (indicated P.Female, Table 1) but accurately matched those of parallel LC-MS analyses which indicated male individuals (Figure 3C, representative examples), thus correcting osteological uncertainties. Comparison of values for endogenous AMELX detection in the tooth extracts, with those for 250pg synthetic peptide standards on the same ELISA plates, indicated typically low nanogram levels of AMELX per well across the eight tooth samples analyzed (not shown).

**Table 1:** Summary table showing antibody-based distinguishing of female from male post-medieval samples matches parallel sex determinations on the same samples by LC-MS or standard osteology on the associated skeletons

Sample	Osteology	LC-MS	ELISA (X/Y)
LU1 (12.5%)	Male	Male	1.75
LU2 (12.5%)	Male	Male	1.96
LU5 (12.5%)	Female	Female	3.28
LU6 (12.5%)	Male	Male	2.00
LU3 (6.25%)	P.Female	Male	3.00
LU4 (6.25%)	Male	Male	2.02
LU7 (6.25%)	P.Female	Male	4.28
LU8 (6.25%)	Female	Female	10.56

P.Female = probably female. Bracketed figures for Sample indicate fraction of total sample loaded per well.

ELISA analyses X/Y values shown, performed blinded to LC-MS or osteology.

**Figure 4:**
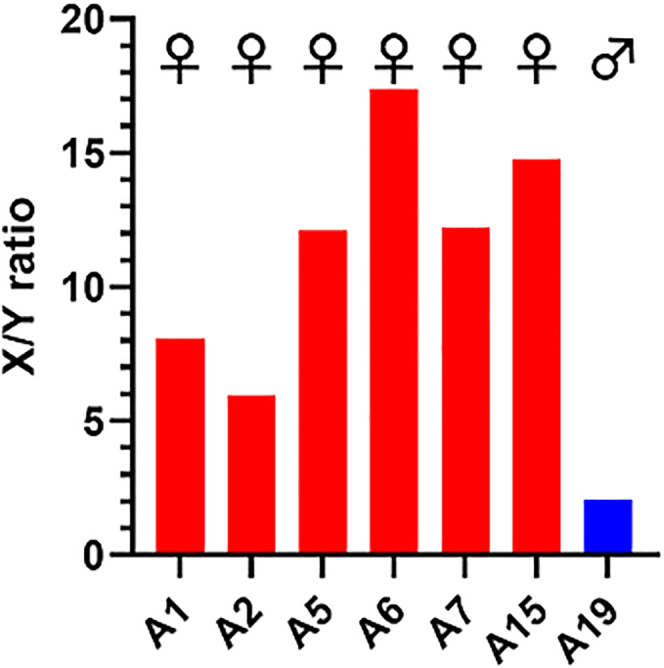
Sex determination of Bronze Age human teeth using manual ELISA AMELX/AMELY (X/Y) ratios detected by ELISA for enamel extracts from seven Bronze Age human enamel samples with “high” (>100pg) endogenous AMELX detection (12.5% of each total enamel extract loaded per well, in duplicate). Male or female assignments, based on ratios, are indicated.

We extended tooth enamel analyses to a selection of more poorly preserved samples from a Bronze Age collection (BCE ∼1600-800, full details to be published elsewhere). For most samples only dental enamel was present and standard osteology was not possible. Samples (typically enamel powder, 2-5mg) were subsampled as for the post-medieval teeth, with 12.5% of the total enamel extract loaded per well. ELISA detection indicated overall lower levels of endogenous AMELX and AMELY recovery than for the post-medieval teeth samples, based on comparison with values for 250pg peptide standards. For quality control, based on our limit of detection ELISA (Figure 2A), here we excluded any test samples where the ratio of endogenous AMELX detection, to 250pg synthetic peptide standard detection on the same ELISA plate, was less than 0.4, i.e., where test samples contained less than ∼100pg endogenous AMELX signal per well. With this criterion applied, 8/15 test samples with “low” AMELX detection were excluded (not shown). For the remaining 7/15 samples with “high” AMELX, the ELISA-based sex determinations (Figure 4) distinguished male and female samples, matching findings of parallel LC-MS analyses (Table 2). With these Bronze Age samples, X/Y ratios for female teeth (which predominated in the collection) were generally higher than for the post-medieval samples, with variability in X/Y ratios presumably reflecting variability in non-specific binding of α-AMELY. For the single male Bronze Age sample (A19) with “high” AMELX detection, in this case an intact tooth, under these conditions, X/Y ratio was notably lower than for the six female samples analyzed, allowing it to be easily discriminated.

**Table 2:** Summary table showing antibody-based distinguishing of female from male Bronze Age samples matches parallel sex determinations on the same samples by LC-MS

Sample	LC-MS	ELISA (X/Y)
A1	Female	8.09
A2	Female	5.97
A5	Female	12.12
A6	Female	17.38
A7	Female	12.23
A15	Female	14.77
A19	Male	2.07

ELISA analyses X/Y values shown, performed blinded to LC-MS.

To determine if observed X/Y ratios were statistically significant between male and female samples from these manual ELISAs (post-medieval and Bronze Age samples combined), distribution of X/Y ratios was determined using Shapiro-Wilks test and found to be significant, p = 0.0049 (i.e., non-normally distributed), thus satisfying the criteria for a two-tailed Mann-Whitney U-test comparing medians between the groups rather than the mean X/Y responses. Median X/Y ratios between male and female groups were 2.02 and 11.34, respectively, with the distribution between the groups statistically significant (*U* = 1, *n*_1_ = 7, *n*_2_ = 8, p = 0.0006), see Figure 6A.

**Figure 5:**
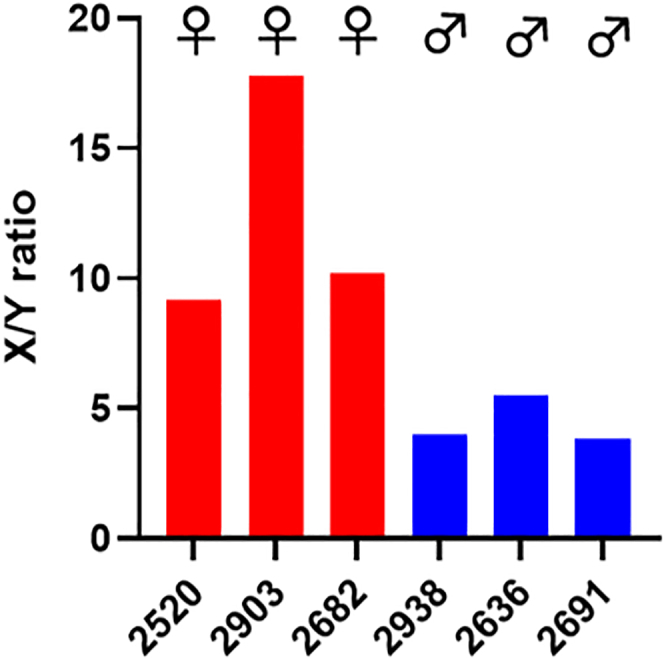
Sex determination of 19^th^ century human teeth using automated ELISA AMELX/AMELY (X/Y) ratios detected by ELISA using automated stations for enamel extracts from six 19^th^ century human enamel samples (12.5% of each total enamel extract loaded per well, in duplicate). Male or female assignments, based on ratios, are indicated.

Finally, toward improved reproducibility and throughput of the ELISA assays and bypassing the need for MS analyses, we applied automated stations to analyze 19th-century teeth, where the sex of the “known” individuals is documented in parish records and identities recorded in ledgers or on headstones. Here, seven teeth were initially selected and analyzed by ELISA using a liquid handling robot and automated plate washer. Again with 12.5% of the total enamel extract loaded per well, robust recovery and detection of endogenous AMELX and AMELY peptides was noted, with X/Y ratios for 6/7 samples consistent with assignment of biological sex from documented records (Figure 5; Table 3). For these teeth, X/Y ratios were more variable and generally higher for males than for the older teeth (post-medieval, Bronze Age) analyzed, which we presume to represent a combination of reduced environmental oxidation of the AMELY peptide for more recent samples, and also the increased dilution of the α-AMELX antibody introduced as part of protocol optimization for robotic application. That aside, the data suggest that under these conditions a cut off X/Y ratio of 8 can be used to distinguish female (above 8) and male (below 8) samples. For the single sample where X/Y ratio was discordant with documented records (1193, ratio ∼10.9 but records indicating male, data not shown), analysis of excavators’ reports subsequently indicated that for this grave, there was uncertainty over the details of the “named” individual exhumed such that individual may have been originally misidentified.

**Table 3:** Summary table showing antibody-based distinguishing of female from male 19^th^ century samples matches documented records of the same samples

Sample	Records	ELISA (X/Y)
2520	Female	9.14
2903	Female	17.78
2682	Female	10.18
2938	Male	4.00
2636	Male	5.50
2691	Male	3.86

ELISA analyses X/Y values shown, performed blinded to documented records.

**Figure 6:**
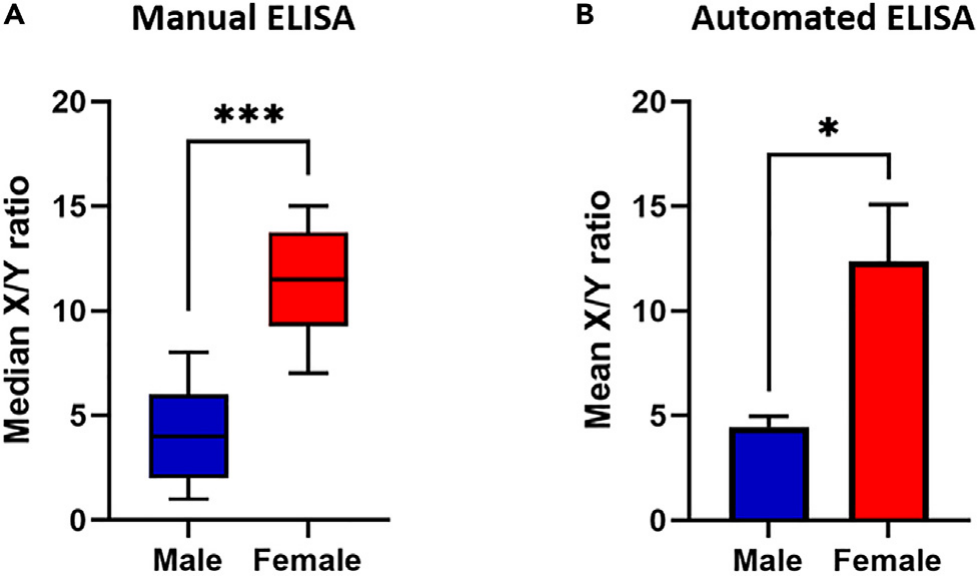
Statistical analyses of ELISA data (A) Boxplot showing median differences between AMELX/AMELY (X/Y) ratios determined using manual ELISAs (data for post-medieval and Bronze Age teeth samples combined). ∗∗∗, p ≤ 0.001, two-tailed Mann-Whitney U-test. Data are represented as median with error bars indicating 5–95 percentile. (B) Bar graph showing differences in mean AMELX/AMELY (X/Y) ratios determined using automated ELISA (19^th^ century teeth samples). ∗, p ≤ 0.05, two-tailed t-test. Data are represented as mean ± SEM.

As for manual ELISAs, statistical analyses confirmed significance in X/Y ratios between the six samples where determined biological sex (ELISA) matched assigned biological sex from documented records. Here, Shapiro-Wilks test found the data to be normally distributed (p = 0.2061) with a two-tailed t-test confirming a difference in mean X/Y ratios (p = 0.0462) between male and female, see Figure 6B.

## Discussion

In summary, we provide a proof-of-concept that antibody-based sex determination, based on detection of endogenous AMELX and AMELY enamel peptides, could in the future serve as a convenient and accessible adjunct or alternative to more specialist MS-based approaches. Collectively these MS and now antibody-based approaches offer exciting possibilities for sex-specific cultural interpretations of not only adult but also juvenile and infant remains, in the absence of major skeletal identifiers, in the archaeological and contemporary forensic and medico-legal science contexts.^9^ Sequence differences in AMELX and AMELY also extend to non-human taxa, indicating the possibility of similar antibody-based approaches to determine sex of other species.

For mainstream adoption of our human antibody-based approach there is clearly a need for further optimization of endogenous AMELX and AMELY detection from archaeological samples with lower peptide recovery, such as the 8/15 Bronze Age samples with “low” AMELX detection that were excluded from our analysis. Those eight samples, largely male (subsequently determined by LC-MS), were recovered from a burial mound and showed poorer sample preservation, accounting for the apparent female bias in the seven samples that were successfully analyzed by ELISA. This need for high sensitivity detection is especially relevant for AMELY peptide, as the Y chromosomally encoded *AMELY* gene and presumably gene product shows expression levels of only ∼10% of *AMELX*.^14^ Indeed, in its current format, inclusion both male and female “standards,” ideally calibrated by LC-MS, is preferable, to aid the male/female discrimination that is possible when AMELX detection is “high.” However, signal amplification techniques, improved (e.g., monoclonal) antibodies, and even alternative peptide detection reagents e.g., affimers, are likely to be able to overcome these caveats. Notably, the move from manual ELISA to use of automated stations did reduce low-level non-specific binding of (α-AMELY) antibodies to components of the enamel extracts evident when probing female samples, indicating future scope for optimizing the assay further.

Indeed antibody-based detection has previously been considered in the archaeological context, for example in the detection of ancient microbes in coprolites,^15^ ancient silks,^16^ and wools.^17^ Although MS based methods continue to make major contributions to the field of palaeoproteomics, for example with the recent development of SPIN (Species by Proteome INvestigation), a high throughput pipeline for species identification from archaeological bone,^18^ our work shows, for the first time that dental enamel, the most durable of human tissues, is an appropriate source of ancient protein antigens for detection by antibody-based approaches.

Our analyses of the 19^th^ century teeth, where the sex of the “known” individuals from which the analyzed teeth were taken was documented in parish records and identities recorded on memorial stones, show that automated stations for moderate/high-throughput analysis of samples is certainly feasible, raising the possibility of low-cost population-level analyses in the future. Analysis of these samples also reinforced the challenges and realities of (osteo) archaeology, with our molecular analysis prompting the realization that one individual may have been originally misidentified. Validated, unbiased molecular approaches, offer opportunities to minimalize such uncertainties in the future.

The use of immunological approaches also offers opportunities for quantitative detection of ancient peptides, as illustrated by our ELISA analysis of synthetic peptide mixtures (Figure 2B) which accurately distinguished different ratios, indicating the potential to probe rare cases of chromosomal abnormalities. While theoretically, karyotypes with multiple copies of the sex chromosomes e.g., aneuploid 47, XXY or 47, XYY, might be probed using antibody approaches, *AMELX* did not appear to be differentially expressed in the different sex-chromosomal aneuploidies when mRNA microarray and qRT-PCR analysis was performed on 471 samples with all possible combinations of X and Y ratios.^19^ In contrast, *AMELY* appears to be dose dependent on the number of Y chromosomes present,^20^ although *AMELY* deletion can occur in phenotypically normal males, in particular in Indian populations.^21^ Clearly further work is needed to differentiate sex chromosome karyotypes using antibody-based (or quantitative MS) approaches, in order to contribute to studies of the natural history of human chromosomal abnormalities.

With the development of technologically advanced and high sensitivity MS-based approaches for bioarchaeology and forensics, antibody-based alternatives may intuitively be viewed as a step backward. However, we reason that such scientific minimalism can help overcome some of the current limitations of MS approaches regarding accessibility. Immunological methods can be cost-effective and rapid, as evidenced from the routine use of lateral-flow tests during the Covid-19 pandemic. In the context of our work, they offer opportunities to make sex determination genuinely a routine activity, applicable to archaeological, museum and teaching collection, without the need for specialist equipment, potentially even in a field setting at a population level, even extending to disaster and war grave scenarios. As an example, Wang and co-worker have previously described a crude immunochromatographic strip assay for detection of ancient wool.^17^ Comparable approaches for sex determination, have the prospect of revolutionizing archaeological and anthropological practice.

### Limitations of the study

Uncertainties related to osteological assessment are well recognized. Polyclonal antibodies generated appear to show limited cross-reactivity against biological samples (although not synthetic peptide antigens) evidenced by AMELY values above zero in enamel extracts from teeth that are known to be female. For the single 19^th^ century tooth where ELISA assessment was discordant with documented records, but subsequent analysis of excavators’ reports indicated that for this grave, there was uncertainty over the details of the “named” individual exhumed, confirmatory MS was not performed as the intended purpose of that analysis was to bypass the need for correlative MS.

## STAR★Methods

### Key resources table

**Table:** 

REAGENT or RESOURCE	SOURCE	IDENTIFIER
**Antibodies**		

Rabbit anti-human AMELX antibodies	Eurogentec	This study
Rabbit anti-human AMELY antibodies	Eurogentec	This study
Peroxidase swine anti-rabbit	Dako	Cat#P0399

**Biological samples**		

Human teeth	Detailed in Experimental model and study participant details section	N/A

**Chemicals, peptides and recombinant proteins**		

Sulfolink® Immobilization Kit for Peptides	Thermo Scientific	Cat#10710825
Enhanced Western Lightning ECL	Perkin Elmer	Cat# NEL103E001EA
1-step ultra TMB ELISA reagent	Thermo Scientific	Cat#10647894

**Other**		

Pierce C18 resin-loaded spin columns	Thermo Scientific	Cat#11814141
NUNC Immuno 96 well plates	Merck	Cat#TMO436110

### Resource availability

#### Lead contact

Further information and requests for resources and reagents should be directed to and will be fulfilled by the lead contact, Rob Layfield (Robert.layfield@nottingham.ac.uk).

#### Materials availability

Requests for access to α-AMELX and α-AMELY antibodies should be directed to the lead contact.

### Experimental model and study participant details

#### Human teeth

Sample size estimations were not relevant. Other procedures performed on the tooth samples (beyond the analyses presented here) are detailed in the following sections.

##### Post-medieval samples

Post-medieval (1500-1850 CE) samples originate from Arnhem, the Netherlands. Arnhem was an industrial centre during this period, specialising in tobacco production, shoemaking, and other factory trades.^22^ Skeletal remains outside of Sint-Eusebiuskerk were excavated in 2007 by the company RAAP and securely dated to 1626-1829 CE *via* church records.^23^ Estimation of sex followed standard osteological analysis of pelvic and cranial features.^24^^,^^25^ A total of eight teeth from eight individuals were sampled for analysis. Premolars and canines were preferentially sampled due to their excellent preservation as well as relative ease of extraction. Based on this osteological analysis, four individuals were assessed as male, two as probably female, and two as female (Table 1). Whole teeth were dry brushed before analysis.

##### Bronze age samples

During archaeological research near the city of Tiel (project Medel De Roeskamp), the Netherlands, in 2016-2017, three Bronze Age burial mounds (BCE ∼1600-800) were found, revealing the remains of at least 18 inhumed and 26 cremated individuals. An Early Bronze Age flat grave cemetery, consisting of four individual inhumation graves and one collective grave with the remains of at least 20 inhumed individuals, was excavated nearby. Unfavourable soil conditions led to poor preservation of skeletal material, including the normally robust dentition. In most individuals, the burial environment fully destroyed all dentine, including the pulp cavity, leaving a hollow and often thin-walled enamel crown (cap). Due to these degradation issues, biological sex was not determined for most samples analysed in this study, except for A7 (Female) and A19 (probably Male). Individuals A2 and A15 were non-adults (10-18 and 5-9 years respectively). The remaining five individuals where data is presented in this study were adults. The delicate teeth were carefully cleaned with Milli-Q water to remove all contaminating soil or loose dentine particles and dried overnight at room temperature. Next, the outer surface of the dental enamel samples was carefully cleaned using an acid–cleaned (Milli-Q – 10% HCl – Milli-Q – ethanol) diamond tipped burr to remove discoloured and potentially diagenetic altered enamel. Circa 4 ± 2 mg of crispy white dental enamel powder was sampled and collected in clean glass vials. After subsamples were taken for Sr-O isotope analysis, the remaining sample material was subject to enamel peptide extraction.

##### 19^th^ century samples

Parishioners were interred at the 19^th^ century cemetery site of St Peter’s Churchyard, Blackburn, UK, from 1821. By the 1860s the cemetery was largely closed to new burials. Blackburn has been a centre of textile production since the 13^th^ century CE. In the early to mid-19^th^ century, however, there was a rapid growth of the cotton industry and a large increase in numbers of looms and factories, largely brought about by linkage to the Leeds-Liverpool canal network in 1810 and the subsequent arrival of the railway in the 1840s. This unprecedented industrial growth drove a ten-fold increase in population, recorded in the 1801 and 1911 censuses. Largely unplanned and unregulated, the expansion of Blackburn resulted in overcrowding and poor sanitation – epidemic disease, high morbidity and mortality were the inevitable consequence.

In 2015, during the course of improvement works to Blackburn town centre, the remains of over 1900 individuals who had been interred at St Peter’s were exhumed by Headland Archaeology (Edinburgh) for reburial in a part of the cemetery unaffected by construction. The excavation of the 19^th^ century burials provided an opportunity to investigate the relationship between social inequalities and health in this provincial, industrialising population. Samples were taken from a representative proportion of the population (n=200) for biomolecular analyses including dietary reconstruction through stable isotope analysis and palaeopathology through amplification and sequencing of pathogen aDNA. Among those individuals sampled were a small number for whom demographic information, including biological sex, was documented in parish records (and was consistent with osteological findings). For this study, teeth from seven individuals of known biological sex were selected (Table 3 for details of six of these). These teeth were cleaned by ultrasonication for 20 minutes in Milli-Q purified water. No other pre-treatment procedures were carried out on these samples.

### Method details

#### Enamel peptide extraction

Tooth enamel peptide extraction and analysis was essentially according to^9^ with enamel etching in 5% (v/v) HCl extended to 60 minutes.^26^ All samples (whole teeth or enamel powder) were briefly washed in 3% (v/v) H_2_O_2_ in a bijou tube for 30 seconds before immediate processing. For whole teeth, etching was achieved by dipping in 200μL of HCl, and for enamel powder typically 2-5mg of enamel was etched in 200μL HCl. Peptides were purified using Pierce C18 resin-loaded spin columns, with the final 60% acetonitrile/0.1% formic acid elution lyophilised.

#### Antibody generation and purification

Synthetic peptide antibodies against AMELX (sequence SIRPPYPSY) and AMELY (sequence SM[Ox]IRPPY, where M[Ox] represents oxidised methionine) were raised in rabbit with standard 87-day protocol (Eurogentec). An additional C-terminal Cys residue allowed coupling of peptide to carrier protein (keyhole limpet haemocyanin) prior to immunisation. For affinity depletions or purification of crude antisera, synthetic peptides were bead immobilised using the SulfoLink® Immobilization Kit for Peptides (Thermo), with 3mg peptide coupled to 2mL beads. For affinity depletion, 20mL of crude antisera made up to 40mL with PBS was rotated with 2mL of beads loaded with the ‘wrong’ synthetic peptide (e.g., α-AMELX *vs* AMELY peptide) for 60 minutes. Depleted antisera were retained and subject to affinity enrichment, by subsequent rotating with 2mL of beads with the ‘correct’ synthetic peptide (e.g., α-AMELX *vs* AMELX peptide). After extensive washing, bound antibodies were eluted (2mL Elution Buffer), neutralised (100μL Neutralization Buffer), dialysed versus PBS, centrifuged (13,000g, 10 minutes, 4°C) and stored in aliquots (-20°C).

#### Dot-blot

Different amounts of synthetic AMELX and AMELY peptides (1-100ng) dissolved in PBS were manually spotted onto nitrocellulose membranes, alongside a mixture of 50ng of each peptide. After drying, membranes were blocked in 3% (w/v) BSA in TBS-Tween (0.05% (v/v)) for 2 hours, then probed with affinity purified α-AMELX or α-AMELY antibodies (1:500 overnight) in blocking buffer. Blots were developed with secondary peroxidase swine anti-rabbit antibodies (Dako, 1:2000 for 2 hours) in blocking buffer with visualisation using ECL (enhanced chemiluminescence, Perkin Elmer Western Lightning).

#### ELISA

For ELISA using synthetic peptides as antigens, peptides (50μL per well in PBS) were loaded into NUNC Immuno 96 well plates in duplicate, along with no peptide control (NPC) wells. Plates were incubated to dryness overnight at 37°C and washed three times with 200μL of TBS-Tween20 (0.1% (v/v)). Plates were subsequently covered and blocked in 5% (w/v) BSA in TBS-Tween for 1 hour at 37°C, then primary antibodies added (1:500 in blocking buffer, 100μL per well, 2 hours at 37°C). Synthetic peptides were routinely detected with both the ‘correct’ primary antibody as well as the ‘incorrect’ primary, in order to assess cross-reactivity. After washing three times in TBS-Tween, peroxidase-conjugated swine anti-rabbit secondary antibodies (Dako) were added (1:2000 in blocking buffer, 100μL per well, 1 hour at 37°C). After washing three times in TBS-Tween, plates were developed using 100μL of 1-step ultra TMB ELISA reagent (Thermo) per well, pre-warmed to room temperature. At the appropriate blue endpoint (2-15 minutes), 100μL of 2N sulphuric acid was added per well to stop the colorimetric reaction. Plates were read at 450nm, with mean NPC values subtracted from mean test values. For analysis of dental enamel extracts as antigen, 6.25-12.5% of total post-medieval teeth extracts (as indicated in Results) were added per well, in 50μL PBS, in duplicate. For the Bronze Age enamel samples, 12.5% of the total enamel extract was loaded per well, in 50μL PBS, in duplicate. For 19^th^ century samples, 12.5% of the total enamel extract was loaded per well, in 50μL PBS, in duplicate, with analysis by automated ELISA (below). For post-medieval teeth, ELISA investigators were blinded against LC-MS and osteology analyses; LC-MS investigators were similarly blinded. For Bronze Age samples, ELISA and LC-MS investigators were blinded against each other and the limited osteology available. For 19^th^ century teeth, ELISA investigators were blinded against documented records. Exclusion of data (Bronze Age samples with low AMELX detection) is indicated in the Results section.

#### Automated ELISA

For the 19th-century teeth the original ELISA was transferred onto an Opentrons OT-2 liquid handling robot (Opentrons LLC, New York) equipped with an 1×1000 µL single channel pipette head. Changes to the original ELISA protocol to optimise the protocol for robotic application were made, consisting of reducing all incubation temperatures to 21°C, increasing the α-AMELX antibody dilution to 1:1000, and adjusting the development time in ultra-TMB to 20 minutes. All TBS-Tween wash steps were performed using an Asys Atlantis automated plate washer (Biochrom, UK), using an 8-channel washhead and a 3x aspirate and fill cycle, 300μL per fill, with a final aspirate cycle for the whole plate. Developed and stopped plates were read on a Biotek Epoch plate reader (Agilent, CA, USA) at 450nm and 560nm (for optical correction of the plate) and the calculated 450-560nm OD reading were then used to subtract the NPC control as before, prior to further analysis.

#### LC-MS analyses

Lyophilised samples (50% of total extracts) were dissolved in 10 μL of 0.1% (v/v) aqueous TFA, centrifuged (100g, 2 minutes), with 8 μL of the supernatant transferred into a tapered LC vial. Samples were analysed with a Dionex U3000 nanoLC coupled to a ThermoFisher LTQ FT Ultra Mass Spectrometer containing a nano-ESI source. 2 μL injection volumes were loaded onto a C18 Pepmap300 loading column (10 mm, 300 Å, 5 μm particle size) with 0.1 % aqueous TFA as a loading solvent. Sample separation used a C18 Pepmap300 column (150 mm × 75 μm, 300 Å, 5 mm particle size) with a gradient of two mobile phases: phase A (5% acetonitrile, 0.1% formic acid) and phase B (95% acetonitrile, 0.1% formic acid). Peptides were eluted using a 30 min linear gradient of mobile phase B from 0% to 55% at a flow rate of 0.4 μL/min followed an increase to 90% B over 0.1 min, held for 5 min before returning to 0% B over 0.1 min, for a column equilibration period of 25 min. The nano-ESI source was operated a voltage of 1.7 kV in the positive ion mode. Entrance capillary temperature was set at 275°C, with inner capillary voltage value set on 37 V and tube lens value of 145 V. Three data channels were acquired. Full scan FTMS spectra were acquired over a 400-1200 *m/z* range at a nominal resolution of 100000 (at *m/z* = 400). MS/MS channels were set to acquire precursor ions at *m/z* 440.2 and *m/z* 540.3, with a window of *m/z* 8, corresponding to the 2+ ions of the AMELY and AMELX peptides, respectively. Precursor ions were fragmented in the ion trap of the spectrometer using He collision gas at a nominal collision energy of 35. Product ions were detected in the ion trap. The instrument was controlled and data visualised by Xcalibur software (Thermo Fisher). The presence of AMELX and AMELY peptides in enamel extracts was determined by plotting chromatograms for the transition *m/z* 540.3 to 714.4 (characteristic for AMELX), and *m/z* 440.2 to 645.4 (characteristic for AMELY).

### Quantification and statistical analysis

Statistical analyses and graphs were performed and generated using GraphPad PRISM (version 9.5.1(733)). Normal distribution of X/Y ratios was determined using Shapiro-Wilks test. Statistical analysis using a two-tailed Mann-Whitney U-test was performed when Shapiro-Wilks test significance was < 0.05 and a two-tailed unpaired t-test was performed when Shapiro-Wilks test significance was > 0.05. A box plot with 5-95 percentile whiskers was chosen for Mann-Whitney U-test to display variance of median X/Y ratios and their overlap; and a bar graph for t-test to display variance in mean X/Y ratios. ∗, P ≤ 0.05; ∗∗∗, P ≤ 0.001.

## Data Availability

• ELISA data for human teeth samples (X/Y ratios, of mean blank corrected anti-AMELX and anti-AMELY values) are presented in Tables 1, 2, and 3. • This paper does not report original code. • Any additional information required to reanalyze the data reported in this paper is available from the lead contact upon request.
